# Towards deployable meta-implants

**DOI:** 10.1039/c8tb00576a

**Published:** 2018-05-16

**Authors:** F. S. L. Bobbert, S. Janbaz, A. A. Zadpoor

**Affiliations:** a Department of Biomechanical Engineering , Delft University of Technology , Mekelweg 2 , Delft 2628CD , The Netherlands . Email: f.s.l.bobbert@tudelft.nl ; Tel: +31-15-2786780

## Abstract

Meta-biomaterials exhibit unprecedented or rare combinations of properties not usually found in nature. Such unusual mechanical, mass transport, and biological properties could be used to develop novel categories of orthopedic implants with superior performance, otherwise known as meta-implants. Here, we use bi-stable elements working on the basis of snap-through instability to design deployable meta-implants. Deployable meta-implants are compact in their retracted state, allowing them to be brought to the surgical site with minimum invasiveness. Once in place, they are deployed to take their full-size load-bearing shape. We designed five types of meta-implants by arranging bi-stable elements in such a way to obtain a radially-deployable structure, three types of auxetic structures, and an axially-deployable structure. The intermediate stable conditions (*i.e.* multi-stability features), deployment force, and stiffness of the meta-implants were found to be strongly dependent on the geometrical parameters of the bi-stable elements as well as on their arrangement.

## Abstract 2

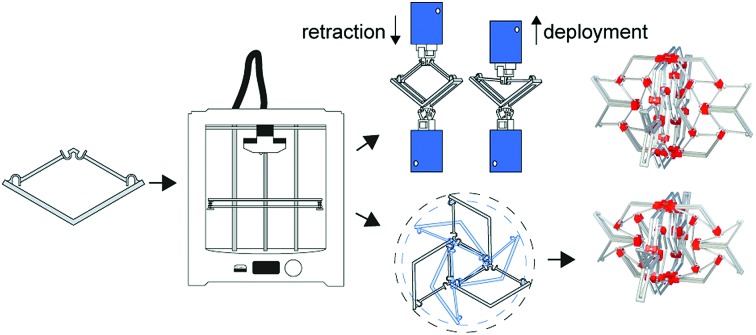
The first step towards deployable meta-implants: design, manufacturing and proof of concept.

## 

We have recently proposed^1^ the concept of meta-implants as orthopedic implants that exploit the rare or unprecedented properties of meta-biomaterials^2^–^4^ to improve their performance and longevity. For example, hybrid meta-biomaterials with a rational distribution of both negative and positive Poisson's ratios could be used to improve bone-implant contact and potentially its longevity.^1^ The unusual properties of meta-biomaterials, in turn, originate from their small-scale (*i.e.* micro/nano-scale) geometrical design. In that sense, meta-biomaterials are similar to other types of designer materials such as mechanical^5^–^9^ or acoustic^10^–^13^ metamaterials.

Here, we propose a new type of meta-implants called ‘deployable meta-implants’. Deployable implants are undersized in their compact mode, which allows them to be brought to the surgical site with a smaller incision and minimum invasiveness. Once they are in place, an activation mechanism deploys the implants into a full-size load-bearing shape. Moreover, deployable implants are fully porous to allow for bone ingrowth.

The main mechanisms used here for the development of deployable implants are the mechanical concepts of bi-14–16 and multi-stability^17^–^19^ that are, for example, seen in snap-through instability systems. Bi-stable structures are part of instability-based metamaterials^20^ and are often based on a snap-through mechanism which enables their structure to shift between two different stable equilibria.^21^,^22^ Due to the existence of two stable equilibrium states, no external forces are required to maintain the structural configuration once it is configured in one of those two positions.^21^,^23^,^24^ By combining bi-stable structures, it is possible to develop multi-stable structures which have more than two stable equilibria.^23^–^29^ In contrast to structures with only one stable or rigid configuration, these structures could adapt their configuration to specific situations.^24^ Two important properties of bi- and multi-stable structures are their capability to be deployed and to absorb energy.^29^ Bi-stable and multi-stable structures could therefore be used in the design of space frame structures,^29^ actuators,^30^ energy absorbing materials,^31^ and energy harvesters.^32^,^33^ For biomedical applications, the concept of multi-stable stents^34^ has been presented before for cardiovascular applications.

The basic elements and assembled multi-stable structures developed in this study are the first step towards deployable structures for application as bone implants. We designed two types of basic bi-stable elements with single curved (D1) and double curved (D2) side hinges where the joints at the center are similar (Fig. 1a). The basic bi-stable elements are composed of flexible components which act as joints and rigid components that fulfill structural functions. Several design parameters including the length (*L*) [mm], angle (*α*) [°], and width (*w*) [mm] (Fig. 1b) determine the mechanical and bi-stable behaviors of D1 and D2. There are at least four different ways of connecting the basic bi-stable elements (T1, T2, T3, T4) to create more complex (multi-stable) mechanisms (Fig. 1d and e).

**Fig. 1:**
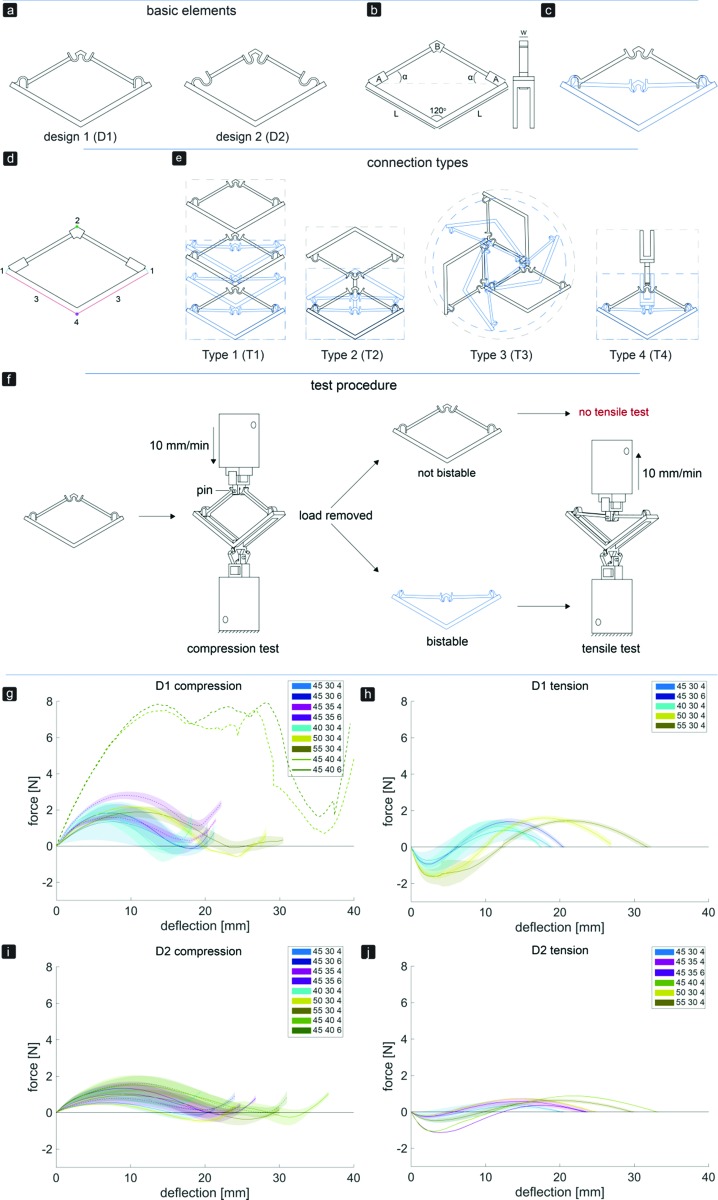
Overview of the two basic element designs. (a) Two basic bistable element designs, D1 and D2, (b) parameters of the basic element, (c) working mechanism of a basic element, (d) connection sites for assembly, (e) different connection types, type 1 (T1) and type 2 (T2): 2D assembly of the basic bistable elements which reconfigures axially, type 3 (T3): 2D assembly of three bistable elements changes dimensions radially, type 4 (T4): 3D assembly which reconfigures axially. The black and blue lines represent the deployed and retracted configurations, respectively. (f) Testing procedure and setup of both compression and tensile tests. A pin at the top of the basic element ensured that both compression and tensile forces were measured at all times. (g–j) Force–deflection diagrams for all variants of bistable element design 1 (g and h) and design 2 (i and j) with different values of parameters *L* [mm], *α* [°], and *w* [mm], under compression (g and i) and tension (h and j).

We performed a parametric study (parameters listed in Table 1) to evaluate the effects of different parameters on both types of behaviors. The design variants were named according to the value of the examined parameters. For example, specimens made according to D1 with *L* = 40 mm, *α* = 30°, and *w* = 4 mm were referred to as D140304.

**Table 1:** Different variants of two basic bi-stable element designs with varying values of dimensions *L*, *α*, and *w*

Design 1	*L* [mm]	*α* [°]	*w* [mm]	Design 2	*L* [mm]	*α* [°]	*w* [mm]
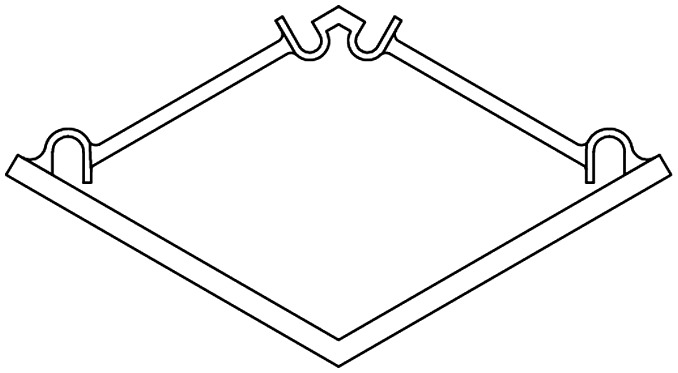	40	30	4	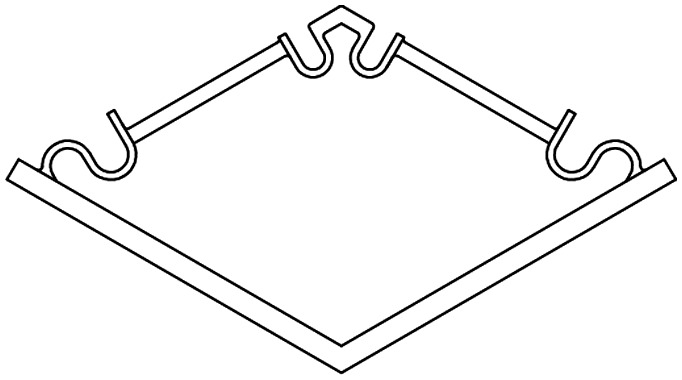	40	30	4
	45	30	4		45	30	4
	50	30	4		50	30	4
	55	30	4		55	30	4
	45	35	4		45	35	4
	45	40	4		45	40	4
	45	30	6		45	30	6
	45	35	6		45	35	6
	45	40	6		45	40	6

3D printers working on the basis of fused deposition modeling (FDM)^35^ (Ultimaker 2+, Geldermalsen, The Netherlands) were used to fabricate the bi-stable (and later multi-stable) structures. A biocompatible polymer, namely poly(lactic acid) (PLA), was used as the main material for printing the basic elements as well as the connecting parts (Fig. 2f) which connected the bi-stable elements for the assembly of the multi-stable structures. In addition to being a biocompatible polymer,^36^,^37^ PLA is biodegradable.^37^,^38^ It has also been proven to be a suitable material for implants onto which cells could adhere and grow.^36^–^38^ The connecting parts were designed in such a way that the proposed connection types could be assembled, and that these small assemblies could be connected together. A mechanical testing machine (Lloyd LR5K) was used to measure the minimum forces required to make the elements shift from their deployed or retracted configuration to their other stable configuration (*F*_S_). To make a distinction between these forces for the compression and tensile tests, (*F*_Sc_) and *F*_St_ were used, respectively. Also the minimum forces required to switch the configurations back to their configurations at the start of the test (*F*_SB_) for both compression (*F*_SBc_) and tensile tests (*F*_SBt_) were evaluated.^39^ All compression and tensile tests were performed at a deformation rate of 10 mm min^–1^. Since the elements were printed in their deployed state, the elements were first tested under compression. When the element was bi-stable after the load was removed, the sample was also tested under tension (Fig. 1f). For the basic elements whose *F*_Sc_ was below 5 N, a 5 N load cell was used. A 100 N load cell was used for the mechanical tests of the assemblies and the basic elements whose *F*_Sc_ was above 5 N. In order to control the direction of the load and to measure the load at all times, extra parts were designed and printed in a similar way to the bi-stable elements (Fig. 2f). All mechanical tests reported in this study were repeated at least three times, unless the design failed at the first trial. The mechanical tests were terminated after the force–deflection curve intersected the *x*-axis for the second time.

**Fig. 2:**
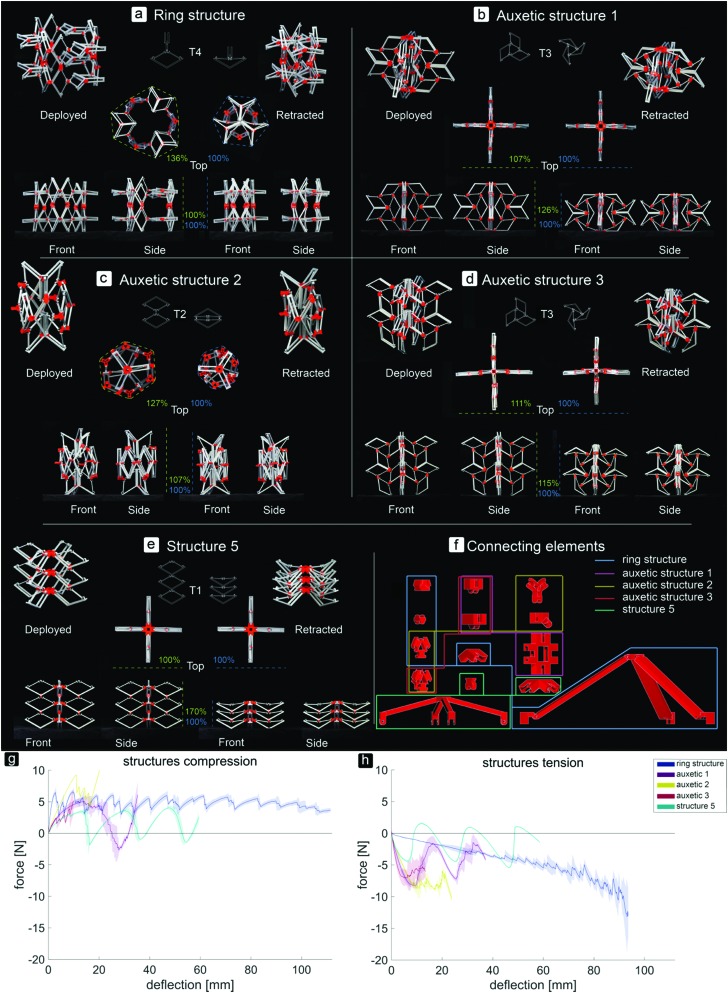
Pictures of different 3D assemblies in their fully deployed and retracted stable configurations. (a) Ring structure, deploying and retracting radially. (b–d) Auxetic structures, retracting upon compression in all directions and deploying upon tension. (e) Structure 5, axially deployable and retractable. (f) Connecting elements to assemble the deployable structures. The elements surrounded by one colour are used for the assemblage of the corresponding structure. The large parts were connected to the top of structure 5 and to both the top and bottom of the ring structure during the tensile and compression tests to enable deformation of the structures. (g and h) Force–deflection curves of the different multistable structures. (g) Compression tests. (h) Tensile tests.

Changing the parameters *L*, *α*, and *w* affected the *F*_S_ and *F*_SB_ values, which are the maximum and minimum forces in the force–deflection graph, respectively (Fig. 1g–j and Table 2). The mean values of the equilibrium paths are shown in the force–deflection curves of the different designs (Fig. 1g–j). All variants showed typical force–deflection curves for the bi-stable structures. These curves start and end with regions showing a positive stiffness, which are separated by a region with a negative stiffness (Fig. 1g–j). It was found (data not shown) that *F*_Sc_ reduces from the first shape shifting cycle to about the third one, and that the force–deflection curves become more constant afterwards. The curves of both D1 and D2 showed a small increase in the *F*_Sc_ and *F*_St_ values when the width of the elements increased from 4 to 6 mm and the corresponding deflection to reach the *F*_Sc_ and *F*_St_ shifted to the right (Fig. 1g and h). The stiffness, amount of deflection, *F*_Sc_, and *F*_St_ were affected by the values of *α* and *L*. Increasing *α* led to a higher stiffness, a higher *F*_Sc_ and *F*_St_, and more deflection of the elements (Fig. 1h), while an increase in *L* led to a slightly lower initial slope during compression. For the four variants, D145354, D145356, D145404, and D145406, *F*_SBc_ was positive (Fig. 1g and Table 2). As for D2, where the side joints consisted of two curves, a lower initial slope and lower values of *F*_S_ and *F*_SB_ were found (Fig. 1i and j). In contrast to the D1 variants, the deflection of the D2 variants reduced when the width increased from 4 to 6 mm (Fig. 1j). For the three variants of D2, *i.e.* D240304, D245306, and D245406, *F*_SBc_ was positive (Fig. 1i). The positive values of *F*_SBc_ found for the variants of D1 and D2 agreed with the observation that these elements were not bi-stable.

**Table 2:** Values of the switching forces of the compression tests (*F*_Sc_ and *F*_SBc_) and tensile tests (*F*_St_ and *F*_SBt_) determined for all the variants of the bi-stable elements D1 and D2 from the force–deflection diagrams

Sample			Design 1								Design 2							
*L*	*α*	*w*	Compression				Tension				Compression				Tension			
			*F*_Sc_	SD	*F*_SBc_	SD	*F*_St_	SD	*F*_SBt_	SD	*F*_Sc_	SD	*F*_SBc_	SD	*F*_St_	SD	*F*_SBt_	SD
40	30	4	1.854	0.5	–0.199	0.7	–1.35	0.3	1.101	0.5	1.088	0.3	0.527	0.2				
45	30	4	1.341	1.1	–0.147	0.3	–0.729	0.5	0.91	0.5	0.551	0.2	–0.136	0.2	–0.243	0.3	0.326	0.2
45	30	6	1.834	0.5	–0.127	0.2	–0.933	0.1	1.392	0.2	0.792	0.1	0.148	0				
45	35	4	1.247	0.1	0.306	0.2					1.027	0.6	–0.005	0.2	–0.266	0.1	0.571	0.5
45	35	6	2.799	0.2	1.131	0.3					1.330	0.4	–0.110	0.4	–1.130	0	0.360	0
45	40	4	7.512		0.705						1.509	0.6	–0.252	0.4	–1.061	0	0.875	0
45	40	6	7.835		1.625						1.598	0.4	0.015	0.3				
50	30	4	2.154	0.1	–0.585	0.1	–1.441	0.4	1.705	0.3	0.487	0.1	–0.455	0.1	–0.183	0	0.720	0
55	30	4	1.884	0.3	–0.089	0.4	–1.299	0.5	1.685	0.4	0.940	0.3	–0.364	0.3	–0.486	0.1	0.627	0.1

In general, due to the stiffer side hinges of D1, the D1 variants required more force to shift from the deployed configuration to the retracted configuration as compared to the D2 variants. For both designs, higher forces were required to make the structures shift from their deployed configurations back to their retracted configurations than *vice versa*. This could be explained by the energy stored in the deflected members of the basic elements during compression.^25^ Therefore, their retracted states are, as desired, less stable than their deployed ones in which the basic elements were printed. The parametric study showed that the D145304 variant is the most stable in its retracted configuration. We therefore used this design variant for the remaining part of the study.

By assembling the basic bi-stable elements with the different connection types (T1, T2, T3, T4), five different 3D deployable structures were developed (Fig. 2a–e). Among these structures, different ways of deployment and retraction were observed. Two of the multi-stable structures retract and deploy radially or axially and exhibit a positive and zero Poisson's ratio, respectively, while three others behave auxetically (*i.e.* exhibit a negative Poisson's ratio). The ring structure, consisting of elements assembled by a combination of T4 connections, deployed and retracted radially (Fig. 2a). Moreover, three different auxetic structures were developed, where the structures retracted in all directions upon compression (Fig. 2b–d). Deployment occurred when the structures were subjected to tension. In two of these structures, *e.g.* auxetic structures 1 (Fig. 2b) and 3 (Fig. 2d), a combination of T3 connections was used. Auxetic structure 2 was designed by combining the rotated versions of T2 connections. The fifth structure (Fig. 2e) was similar to the T1 connection (Fig. 1e), where deployment and retraction occurred axially in the direction of the applied force (Fig. 3).

**Fig. 3:**
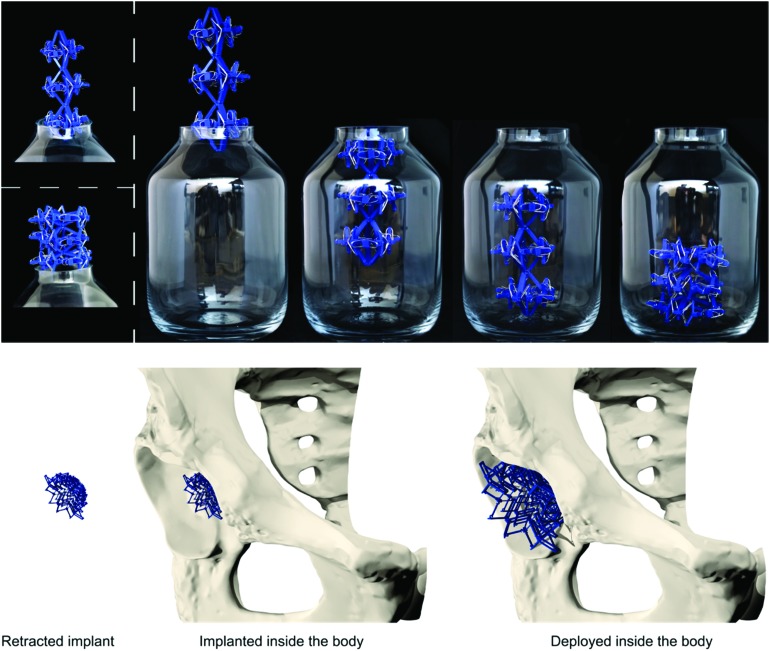
Top: Example of how a multistable structure (based on the ring structure) can be put inside a bottle when the deployed configuration does not fit through the opening. Bottom: Example of how multistable structures could be applied as a bone implant.

The assemblies showed different changes in dimensions (height (*h*), width (*w*), and circumference (*c*)) from the retracted to the deployed configuration (Fig. 2a–e). Structure 5 showed the largest change (*h*: 170%), followed by the ring structure (*c*: 136%), auxetic structure 2 (*c*: 127%, *h*: 107%), auxetic structure 1 (*h*: 126%, *w*: 107%), and finally auxetic structure 3 (*h*: 115%, *w*: 111%). The ring structure required the highest force for complete retraction (Fig. 2h). For this type of structure, additional parts (Fig. 2f) were developed to make the structure deploy or retract when under compression or tension, respectively. The *F*_Sc_ values of the different stable configurations were similar during compression, while *F*_St_ increased during tension (Fig. 2g and h). Auxetic structure 1 showed an increase in load both under compression and tension, followed by one valley or a valley, a peak and a valley, respectively (Fig. 2g and h). The force–deflection curve of auxetic structure 2 during compression started with a linear region with increasing loads up to about 10 N. After this peak, there was a negative slope consisting of three peaks and four valleys (Fig. 2f). Auxetic structure 3 showed two clearly different curves for compression and tension (Fig. 2g and h). While the force peaks of this type of structure increased when it was subjected to compression, the first peak in the curve of the tensile tests was the highest, followed by some lower peaks (Fig. 2g and h). Structure 5 deployed and retracted only in the direction of the applied load and showed three peaks of similar heights and three valleys when subjected to either tension or compression (Fig. 2g and h).

This study showed that by assembling the basic elements in various ways, different multi-stable structures which reconfigure differently could be obtained. Besides two multi-stable structures which deployed and retracted radially or axially, three auxetic structures were developed. In the case of the ring structure, increasing forces were found during the tensile tests as compared to the more equal forces during the compression tests. Upon compression, the ring was pushed outwards which made the elements shift from their retracted configuration to their deployed configuration. The results of the basic elements showed that their retracted configuration is less stable than their deployed configuration, hence the lower forces during the compression of the ring structure. The opposite occurred when the structure was subjected to tension, when the elements had to shift from their deployed state to their retracted state.

When auxetic structure 1 was subjected to tension or compression, the elements gradually snapped into their other stable configurations. As could be seen in the force–deflection graph, the force drops suddenly at some points. At these points of deflection, a combination of several elements in the T3 connection snapped through at the same time.

The force–deflection curves of auxetic structure 2 showed that for both compression and tension, the first force peak was high, meaning that about 10 N was required to make the first snap-through of one of the elements happen within the structure. However, when the first element switched its configuration, the other elements followed shortly after. During both the tensile and compression tests, we observed that the elements reconfigure gradually but quickly after each other, which explains the similar drops in force after the succeeding *F*_S_ were reached.

During both compression and tensile tests of auxetic structure 3, not all the elements retracted or deployed, respectively. It was found that the elements at the sides of the structure deployed first, after which only two of the elements at the top deployed. Finally, three of the four elements at the bottom of the structure switched their configurations. Since the elements at the top of the structure were not connected to the other elements such that they were forced to deploy, they remained in their retracted configuration during the tensile tests.

Structure 5, which deployed and retracted axially, showed three peaks and valleys in both compression and tensile tests. These peaks represent the least amount of force required to switch from one to another stable configuration of this structure. During the compression tests, first the bottom layer retracted, followed by the second layer and finally the top layer. This order was reversed when the structure deployed.

This study showed that multi-stable structures with different deploying and retracting behaviors could be generated based on simple bi-stable elements. It was shown that some of these structures, *e.g.* structure 5, the ring structure, and auxetic structure 1, are capable of shifting between two distinct configurations (deployed and retracted) with some stable configurations in between.

In summary, we described the design and manufacturing process of simple bi-stable elements and their assembly into deployable 3D structures. Different parameters of the bi-stable elements affected not only the force required to make the structure shift from one stable position to the other, but could also lead to elements which were not bi-stable at all. Moreover, energy is stored in the bi-stable elements when they are configured in their retracted state. This resulted in lower *F*_St_ values during deployment as compared to the *F*_Sc_ values during retraction. The multi-stable structures could be deployed and retracted axially, radially, and behave auxetically. Auxetic structures are especially interesting for application as minimally invasive deployable meta-implants. Due to their small dimensions in all directions in their retracted configuration, the size of incision and the damage to the surrounding tissues are minimized during surgery. The recovery time of patients and the chance of post-operative implant-associated infections are therefore expected to be reduced.

The multi-stable structures presented here need to be further developed before actual clinical application. The high porosity of these deployable structures allows for improved bone ingrowth. As deployable implants use the minimum amount of materials, a major design challenge is to ensure that they provide enough mechanical support. Future research should therefore be focused on evaluating the mechanical performance of meta-implants as well as on designing miniaturized versions that make them more suitable for application as bone substitutes.

## Conflicts of interest

There are no conflicts to declare.

## References

[cit1] Kolken H. M., Janbaz S., Leeflang S. M., Lietaert K., Weinans H. H., Zadpoor A. A. (2018). Mater. Horiz..

[cit2] Ahmadi S., Hedayati R., Li Y., Lietaert K., Tümer N., Fatemi A., Rans C., Pouran B., Weinans H., Zadpoor A. (2018). Acta Biomater..

[cit3] Bobbert F., Lietaert K., Eftekhari A. A., Pouran B., Ahmadi S., Weinans H., Zadpoor A. (2017). Acta Biomater..

[cit4] Zadpoor A. A. (2017). Int. J. Mol. Sci..

[cit5] Berger J., Wadley H., McMeeking R. (2017). Nature.

[cit6] Coulais C., Kettenis C., van Hecke M. (2018). Nat. Phys..

[cit7] Hedayati R., Leeflang A., Zadpoor A. (2017). Appl. Phys. Lett..

[cit8] Zadpoor A. A. (2016). Mater. Horiz..

[cit9] Zheng X., Lee H., Weisgraber T. H., Shusteff M., DeOtte J., Duoss E. B., Kuntz J. D., Biener M. M., Ge Q., Jackson J. A. (2014). Science.

[cit10] Bückmann T., Thiel M., Kadic M., Schittny R., Wegener M. (2014). Nat. Commun..

[cit11] Chen H., Chan C. (2007). Appl. Phys. Lett..

[cit12] Fok L., Ambati M., Zhang X. (2008). MRS Bull..

[cit13] Lee S. H., Park C. M., Seo Y. M., Wang Z. G., Kim C. K. (2009). J. Phys.: Condens. Matter.

[cit14] Crivaro A., Sheridan R., Frecker M., Simpson T. W., Von Lockette P. (2016). J. Intell. Mater. Syst. Struct..

[cit15] Silverberg J. L., Na J.-H., Evans A. A., Liu B., Hull T. C., Santangelo C. D., Lang R. J., Hayward R. C., Cohen I. (2015). Nat. Mater..

[cit16] Yasuda H., Yang J. (2015). Phys. Rev. Lett..

[cit17] Harne R. L., Wu Z., Wang K.-W. (2016). J. Mech. Design.

[cit18] Santer M., Pellegrino S. (2011). J. Mech. Design.

[cit19] Waitukaitis S., Menaut R., Chen B. G.-g., van Hecke M. (2015). Phys. Rev. Lett..

[cit20] Bertoldi K., Vitelli V., Christensen J., van Hecke M. (2017). Nat. Rev. Mater..

[cit21] Ohsaki M., Nishiwaki S. (2005). Struct. Multidiscip. Optim..

[cit22] Ha C. S., Lakes R. S., Plesha M. E. (2018). Mater. Des..

[cit23] Oh Y. S., Kota S. (2009). J. Mech. Design.

[cit24] Santer M., Pellegrino S. (2008). Int. J. Solids Struct..

[cit25] Santer M., Pellegrino S. (2011). J. Mech. Design.

[cit26] Chen G., Aten Q. T., Zirbel S., Jensen B. D., Howell L. L. (2010). J. Mech. Robot..

[cit27] Zhang J., Zhang C., Hao L., Nie R., Qiu J. (2017). Appl. Phys. Lett..

[cit28] Restrepo D., Mankame N. D., Zavattieri P. D. (2015). Extreme Mech. Lett..

[cit29] Chen T., Mueller J., Shea K. (2017). Sci. Rep..

[cit30] Gerson Y., Krylov S., Ilic B., Schreiber D. (2012). Finite Elem. Anal. Des..

[cit31] Shan S., Kang S. H., Raney J. R., Wang P., Fang L., Candido F., Lewis J. A., Bertoldi K. (2015). Adv. Mater..

[cit32] Wu Z., Harne R. L., Wang K.-W. (2014). J. Appl. Mech..

[cit33] Arrieta A., Hagedorn P., Erturk A., Inman D. (2010). Appl. Phys. Lett..

[cit34] Kuribayashi K., Tsuchiya K., You Z., Tomus D., Umemoto M., Ito T., Sasaki M. (2006). J. Mater. Sci. Eng. A.

[cit35] Zadpoor A. A., Malda J. (2017). Ann. Biomed. Eng..

[cit36] Ramot Y., Haim-Zada M., Domb A. J., Nyska A. (2016). Adv. Drug Delivery Rev..

[cit37] Kulkarni R., Pani K., Neuman C. and Leonard F., Polylactic acid for surgical implants, Walter Reed Army Medical Center Washington DC Army Medical Biomechanical Research Lab, 1966. 10.1001/archsurg.1966.01330050143023 5921307

[cit38] Gupta B., Revagade N., Hilborn J. (2007). Prog. Polym. Sci..

[cit39] Palathingal S., Ananthasuresh G. (2017). Mech. Mach. Theory.

